# Fatal native aortic valve fungal endocarditis caused by *Aspergillus flavus*: A case report

**DOI:** 10.1016/j.idcr.2021.e01310

**Published:** 2021-10-13

**Authors:** Abdulrahman F. Al-Mashdali, Mohammed A. Alamin, Ammar M. Kanaan, Abdulaziz Alkhulaifi, Dawoud I. Al Kindi

**Affiliations:** a Department of Internal Medicine, Hamad Medical Corporation, Doha, Qatar; b Department of Cardiology, Heart Hospital, Hamad Medical Corporation, Doha, Qatar; c Department of Cardiothoracic Surgery, Heart Hospital, Hamad Medical Corporation, Doha, Qatar

**Keywords:** Endocarditis, Fungal, *Aspergillus flavus*, Diabetes mellitus, Case report

## Abstract

Fungal endocarditis is a rare condition, specifically in immunocompetent patients. Aspergillus species are the etiology in less than 30% of the cases. Moreover, *Aspergillus flavus* endocarditis is extremely rare and reported in only 7% of the total Aspergillus endocarditis cases. The most common predisposing factors are immunocompromised state, prosthetic valve, and previous cardiac surgery. In most cases, the diagnosis is delayed and occasionally missed. Prompt medical management combined with early surgical intervention is recommended once the diagnosis is established since the mortality rate is nearly 100% without surgical intervention. We report a rare and fatal case of native aortic valve endocarditis in a 49 years old diabetic patient who presented with fever and abdominal pain, complicated by multiple septic embolizations (splenic infarction, cerebral emboli, and limbs ischemia), and in which A. *flavus* was confirmed post mortem.

## Introduction

Fungal endocarditis represents around 2% of infective endocarditis cases. Candida species are the major causative organism and account for more than 70% of fungal endocarditis cases [1]. The remaining cases (around 20–30% of fungal endocarditis) are mainly due to infection by Aspergillus species which are the most common cause of intracardiac fungal ball [2]. The most prevalent predisposing factors are a history of immunocompromise status, prosthetic valves, and previous cardiac surgery of the affected valve [3]. The diagnosis and management of Aspergillus endocarditis (AE) are challenging; hence, it carries a poor prognosis and high mortality rate [1], [2], [3]. In extremely rare occasions, AE affects the native valve of immunocompetent individuals [2], [3]. Herein, we report a rare and fatal case of *Aspergillus flavus* endocarditis that affected the native aortic valve in a 49-year-old diabetic patient.

## Case presentation

**Fig. 1:**
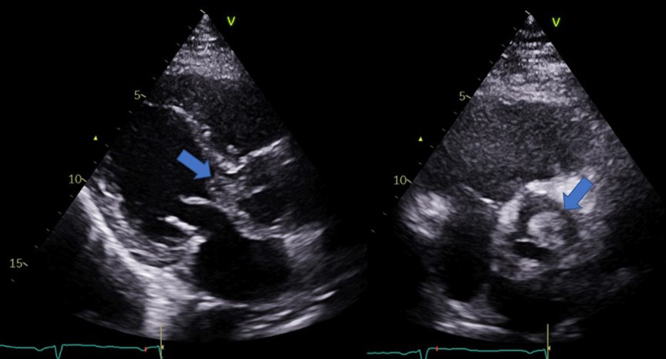
Echocardiography showing a mass (i.e., Fungal ball) attached to the cusps of the aortic valve (blue arrows). (For interpretation of the references to color in this figure legend, the reader is referred to the web version of this article.)

**Fig. 2:**
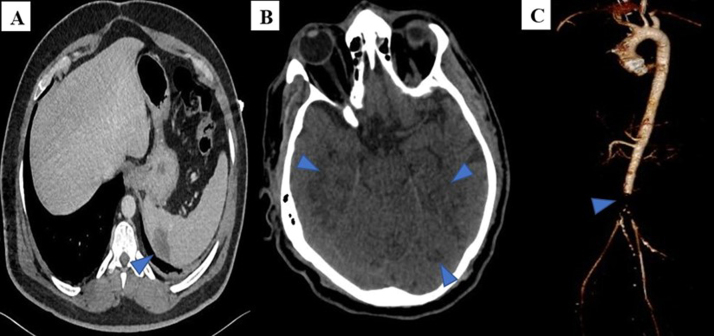
(A) CT abdomen showing wedge-shaped hypodense lesion consistent with splenic infarction, (B) CT brain showing multiple hypodense lesions involving temporal and occipital lobes consistent with infarcts due to showering emboli, (C) CT aortogram depicting occlusion of abdominal aorta bifurcation.

A 49-years-old male presented to the emergency department (ED) with a history of intermittent fever and abdominal pain for one week. He had a history of diabetes mellitus (DM) (HbA1c was 7.2% on oral hypoglycemic medications) and coronary artery disease. One year earlier, he underwent cardiac surgery for mitral valve fibroelastoma resection and coronary artery bypass grafting (CABG). There was no history of smoking, alcohol intake, or substance abuse. On examination, he was afebrile initially, and other vital signs were within the normal range. Abdominal examination revealed tenderness in the left upper quadrant without guarding or rigidity. Examination of the cardiovascular and respiratory systems was unremarkable. Laboratory results were significant for normocytic anemia (hemoglobin was 10.9 g/dL) and elevated C-reactive protein level (147.5 mg/L), but normal renal and liver functions parameters. Abdominal computed tomography (CT) showed a wedge-shaped hypodense lesion in the spleen suggestive of splenic infarction [Fig. 1A]. Echocardiography (echo) revealed moderate aortic regurgitation and a rounded mass measuring approximately 1.5 by 1 cm attached to the left aortic valve coronary cusp, which was not present in the previous echo one year earlier [Fig. 2]. The patient was initiated on empirical antimicrobial therapy. As the patient continued to be febrile in the initial days of hospital admission and blood cultures did not grow any organisms, the antibiotics coverage was escalated to cefepime and vancomycin.

After six days of admission, he developed acute chest pain associated with severe breathlessness. He was tachypneic and tachycardic; he was desaturated to 90%; chest examination revealed bilateral crepitation, so immediate medical management for acute pulmonary congestion was initiated with intravenous diuretics and nitroprusside infusion, and non-invasive ventilation. As his respiratory parameters did not show significant improvement, he was electively intubated. Repeated echo showed severe aortic regurgitation with normal ejection fraction. A decision was made for surgical intervention, and surgical aortic valve replacement was done. Intraoperative findings included the presence of abscess formation at the aortic valve annulus and aortic wall at the level of the sinus-tubular junction, in addition to the destruction of the left coronary leaflet. The mitral valve leaflets were normal in appearance.

Postoperatively, clinical examination revealed unequal pupils and cold extremities. CT brain showed multiple brain hypodensities, suggesting thrombo-embolic stroke [Fig. 2B]. CT aortogram demonstrated saddle thrombus at the aortic bifurcation occluding the proximal parts of common iliac arteries [Fig. 2C]. Subsequently, emergency laparotomy was carried on with successful removal of aortic thrombus, and hemicolectomy was performed due to the presence of gangrenous bowel. On day nine, the fungal stain was positive from the tissue culture of the aortic valve; hence, amphotericin B was added to the antimicrobial regimen. However, the patient's clinical condition deteriorated, and he developed septic shock and multiorgan failure. Unfortunately, on day 11 of admission, the patient had a cardiac arrest and passed away. The following day after the patient's death, the fungal culture of the aortic valve mass revealed A. *flavus*.

## Discussion

Aspergillus endocarditis (AE) is a rare condition that is associated with fatal sequela. More than 60% of the cases are caused by *Aspergillus fumigatus*, whereas A. *flavus* constitute 7% of all AE cases [2]. Historically, the most common risk factors are underlying cardiac abnormalities and the presence of prosthetic valves [3]. However, a recent review article concluded that immunocompromised status, use of central line catheters, and prolonged antimicrobial administration had been the most common predisposing factors [1]. In our patient, the previous cardiac surgery was related to the removal of mitral valve fibroelastoma and surgical revascularization, whereas the native aortic valve was infected. In addition, although he has DM, it is not a well-established predisposing factor [1], [2], [3].

The diagnosis of AE is clinically challenging that is attributed mainly to the non-specific and insidious presentation. The main presenting symptom is fever [2], [3]. Other common symptoms include fatigue, generalized pain, loss of appetite, and symptoms of peripheral embolization. Blood cultures are negative in more than 90% of the cases [2]. Accordingly, in most cases, AE diagnosis is reached by histopathology of the infected cardiac tissue. Moreover, in some instances, the diagnosis of AE is made after the patient's death, similar to our case [4], [5]. Echocardiography (echo) is helpful in the visualization of intracardiac vegetation. Transthoracic echo (TTE) can detect up to 90% of valve vegetations, while transesophageal echo is more sensitive and frequently needed for vegetation quantification and any valvular regurgitations severity assessment [3], [5]. In our case, TTE showed sizeable aortic valve vegetation. In comparison with TTE, Cardiac MRI has a role in quantifying cardiac masses tissue characteristics [6]. Of note, Aspergillus PCR, 1,3-β-D-glucans (BDG), and/or galactomannan (GM) tested on a blood sample may aid in the early diagnosis or the exclusion of AE. A review article that included 20 cases of AE showed that Aspergillus PCR test was positive in all cases, while BDG and GM were positive in 85.7% and 62.5% of cases, respectively. However, all three tests were negative in 20% of the cases [7], [8], [9].

Systemic embolization is encountered in around 75% of AE cases which is related to the usual large vegetation size, high mobility, and fungal ball friability. Our patient developed multiple embolic complications, including splenic infarction, aortic embolism, and embolic stroke. Medical and surgical management should be started simultaneously as early as possible. The mortality rate in medically treated patients alone may approach 100%. However, despite surgical removal of infected cardiac tissue, the mortality rate remains high and is estimated to reach 68% [1], [2], [3]. Infectious Diseases Society of America (IDSA) guidelines recommend combining medical and surgical management strategies to minimize probable cardiac complications and systemic embolization. Based on the latest IDSA guidelines, the drugs of choice for AE treatment are voriconazole or liposomal amphotericin B, followed by a secondary prevention strategy of lifelong antifungal therapy after hospital discharge [10], [11]. Regrettably, our patient passed away due to septic embolization of the fungal ball to several vital organs despite surgical intervention and antifungal coverage.

## Conclusion

Aspergillus endocarditis is a rare and fatal condition that carries diagnostic and therapeutic challenges to the treating physicians. A high index of suspicion and serology test is essential for the early diagnosis. A combination of medical and immediate surgical management strategies is recommended to attain the best outcomes.

## Ethical approval

Ethical approval was obtained from Medical Research Centre (MRC) in Hamad Medical Corporation (HMC).

## Funding

This research did not receive any specific grant from funding agencies in the public, commercial, or not-for-profit sectors.

## CRediT authorship contribution statement

Abdulrahman F. Al-Mashdali: Acquisition of data; Drafting the manuscript; Approval of the version of the manuscript to be published, Mohammed Altayeb: Drafting the manuscript; Approval of the version of the manuscript to be published, Ammar M. Kanaan: Revising the manuscript critically for important intellectual content; Approval of the version of the manuscript to be published, Abdulaziz Alkhulaifi: Revising the manuscript critically for important intellectual content; Approval of the version of the manuscript to be published, Dawoud Al Kindi: Analysis and interpretation of data; Revising the manuscript critically for important intellectual content; Approval of the version of the manuscript to be published.
